# Inequity in Health Services Utilization in Economically Underdeveloped Regions of Northeast China

**DOI:** 10.3389/fpubh.2022.850157

**Published:** 2022-04-15

**Authors:** Xin Zhang, Ning Ning, Hongguo Zhou, Linghan Shan, Yanhua Hao, Mingli Jiao, Libo Liang, Zheng Kang, Ye Li, Huan Liu, Baohua Liu, Kexin Wang, Adelina Ruzieva, Lijun Gao, Qunhong Wu

**Affiliations:** ^1^Department of Health Policy, School of Health Management, Harbin Medical University, Harbin, China; ^2^Department of Social Medicine, School of Public Health, Harbin Medical University, Harbin, China; ^3^Office of Educational Administration, Ningbo College of Health Sciences, Ningbo, China; ^4^School of Health Services and Management, Ningbo College of Health Sciences, Ningbo, China

**Keywords:** healthcare utilization, inequality, inequity, horizontal inequity index, critical illness insurance

## Abstract

**Background:**

The Chinese health system has long been committed to eliminating inequalities in health services utilization. However, few studies have analyzed or measured these inequalities in economically underdeveloped regions in China.

**Methods:**

A total of 6,627 respondents from 3,000 households in Heilongjiang Province were extracted from the Sixth National Health Services Survey. We measured horizontal inequity in both 2-week outpatient rate and annual inpatient rate, and then identified the factors contributing to inequality.

**Results:**

The horizontal inequity indices of the 2-week outpatient and annual impatient rates in Heilongjiang Province were 0.0586 and 0.1276, respectively. Household income, health status, place of residence, basic medical insurance, and commercial health insurance were found to be the main factors affecting inequality in health services utilization. The contributions of household income to these two indices were 184.03 and 253.47%, respectively. Health status factors, including suffering from chronic disease, limitations in daily activities, and poor self-rated health, played positive roles in reducing inequality in these two indices. The contributions of place of residence to these two indices were 27.21 and −28.45%, respectively. Urban Employee Basic Medical Insurance made a pro-rich contribution to these two indices: 56.25 and 81.48%, respectively. Urban and Rural Resident Basic Medical Insurance, Urban Resident Basic Medical Insurance, New Rural Cooperative Medical Scheme, and other basic medical insurance made a pro-poor contribution to these two indices: −73.51 and −54.87%, respectively. Commercial health insurance made a pro-rich contribution to these two indices: 20.79 and 7.40%, respectively. Meanwhile, critical illness insurance made a slightly pro-poor contribution to these two indices: −4.60 and −0.90%, respectively.

**Conclusions:**

The findings showed that the “equal treatment in equal need” principle was not met in the health services utilization context in Heilongjiang Province. To address this issue, the government could make policy changes to protect low-income populations from underused health services, and work to improve basic medical insurance, critical illness insurance, and social security systems.

## Introduction

Equity in health care access and other social amenities are the main tenets of the Universal Declaration of Human Rights (1). Inequality and inequity are major problems in healthcare systems across the world (2–4). Substantial inequalities exist in health and health services utilization. Constraining factors include low income, poor living conditions, and reduced access to health services, all of these make it more likely that poor populations will have diminished self-assessed health (5) and higher rates of both mortality and morbidity when compared to wealthier populations (6–8). In fact, poor populations often access fewer health services despite higher demand (6). Multiple studies report a clear correlation between inequalities in health service utilization and divergent income brackets, the findings suggest that there are higher utilization rates in high-income populations compared with low-income ones (9–11). Rich populations are much more likely to use high-quality health services (12). Moreover, poor individuals often use a comparatively greater percentage of their income on health care, perpetuating poor health and poverty (6). In addition to the general inequalities found between poor and rich populations, inequalities in health services utilization often exist between regions within the same country (3, 13, 14). Many disadvantages exist in rural, remote, and poor areas, where the residents tend to experience disadvantages due to factors such as low income (6, 15, 16).

To tackle inequity in health services utilization, we first need to identify barriers to equity. Horizontal equity is a widely accepted principle in health care inequality research (17). This principle calls for the equal treatment of people who have equal needs, regardless of their income (18). Many studies on horizontal equity have reported that multiple factors can affect health services utilization. For example, several have reported that economic status is the most prominent factor between poor and rich populations (3, 10, 19), with others being health status, medical insurance, and health policies (3, 20, 21). Given these factors, some cases of inequality are substantial and inevitable, as some of the determinants of health services utilization are unavoidable. While there may be ways to alter the inequitable distribution (e.g., basic medical insurance and critical illness insurance), such alteration is often dependent on policymakers. Health insurance appears to be a foremost factor in improving inequality in health care. The Chinese central government aimed at both protecting residents from large medical expenses and improving the universal health insurance coverage by launching critical illness insurance in August 2012 (22). The national implementation date was 2016. Critical illness insurance for urban and rural residents was piloted in the cities of Harbin and Suihua in Heilongjiang Province in 2013 and formally implemented province-wide in 2015 (23). Very few studies in Chinese underdeveloped regions have examined the multiple factors that impact inequality in the use of health services. These include socioeconomic and health status, and health care accessibility, especially when taking critical illness insurance into account. This highlights the need for further research to determine whether critical illness insurance promotes the equitable utilization of health services.

Due to limited aggregated data, some Chinese studies have focused on specific populations, including women (9), patients with chronic non-communicable diseases (10), residents of urban areas (24), and residents of rural areas (25, 26). Besides, some studies have focused on the general population (3, 27). Still, few studies have investigated inequality in health services utilization in economically underdeveloped regions. Research is needed to identify the main barriers to health services utilization equality in these areas. Policymakers and healthcare providers require detailed information to guarantee health services utilization for people living in unfavorable conditions. To fill this research gap, this study sets the three following goals: (1) determine whether the “equal treatment in equal need” principle is being met in the context of health services utilization in economically underdeveloped regions of northeast China, and (2) identify the main barriers to equal utilization, and (3) provide the supporting data for government policy making.

## Materials and Methods

### Data Source

The data used in this study have been obtained from the Sixth National Health Services Survey (NHSS 2018), of Heilongjiang Province in the NHSS 2018. The Sixth NHSS was conducted in 31 provinces in 2018. To ensure a representative sample, using economical and effective sampling criteria, a four-stage stratified random sampling method was adopted by them. In the first stage, 156 counties/cities were proportionally and randomly selected nationwide, of which five counties/cities were randomly selected in Heilongjiang Province. In the second stage, five rural townships or five urban districts were chosen at random in each county/city. In the third stage, two villages or two neighborhoods were randomly selected in each township/district. In the fourth stage, 60 households were randomly selected in each village/neighborhood. All the participants were interviewed face-to-face survey using an electronic tablet by trained investigators. The participants of the survey are the resident population among the sampled households.

Heilongjiang Province is located along the northeast border of mainland China, covering an area of 473,000 km^2^. In 2018, there was a population of 37.73 million living there, 60.10 and 39.90% of residents lived in urban and rural areas, respectively (28). At the end of 2018, the per capita gross regional product of Heilongjiang Province was CNY 43274, thus ranking 27th among the 31 provinces (29). The Sixth NHSS was conducted there in September 2018. After data cleaning, 6,627 respondents were included, with a final household sample size of 3,000.

### Ethics Statement

Ethics clearance was obtained from the Ethics Committee of Harbin Medical University.

### Measurements

#### Dependent Variables

Health services were measured by two indices: (1) use (yes or no) of outpatient care over a 2-week period and (2) use (yes or no) of inpatient care over a twelve-month period. Respondents were asked: “Have you received any medical treatment during the last 2 weeks?” and “Have you been admitted to hospital during the past year?”.

#### Independent and Control Variables

Health services utilization is associated not only with responses to need variables, but also with non-need variables. In this study, need factors included gender, age, self-assessed health, chronic diseases, and limitation of daily activities. Non-need factors included household income, marital status, education, employment status, place of residence, health insurance (social basic medical insurance, critical illness insurance, and commercial health insurance), distance to nearest health facility, and time taken to travel to nearest health facility. Household income was measured using per capita consumption expenditure. We used the EQ-VAS score to evaluate health status in participants, ranging from zero (worst health) to 100 (best health) (30). Consistent with a previous study (3), EQ-VAS scores were divided into five categories according to specific cut-offs (20, 40, 60, and 80).

### Statistical Analysis

The difference in health services utilization with different social demographics was analyzed using the *X*^2^ test and the Cochran–Armitage test. For the unordered categorical variables, such as marital status and employment status, the *X*^2^ test was used. For ordinal variables (age, household income, education, and self-assessed health), the Cochran–Armitage test was used. In this study, we used a concentration index (CI) to measure the degree of household income-related inequality in health services utilization. CI was further decomposed to assess the contribution of different factors (need factors and non-need factors) in explaining inequality in health services utilization. The horizontal inequity (HI) index indicated the household income-related inequity in health services utilization after standardizing for differences in health needs, such as gender, age, and health status. HI was calculated based on the CI decomposition results. These methods were proposed by Wagstaff et al. (31, 32) and extensively used by many Chinese researchers (9, 10, 21, 33, 34). The calculation steps are as follows.

### First Step: Standardization of Health Services Utilization

We calculated the distributions of actual services use, need-expected services use, and need-standardized services use across household income quintiles. Actual services use is a factual depiction of the extent of equality (or inequality) in the distribution of health services. Need-expected services use represents predicted services use based on the “need variables.” Underuse occurs when actual services use is smaller than need-expected services use, while overuse occurs when actual services use is larger than need-expected services use. We used need-standardized services use to reflect inequity, with the aim of determining how the actual distribution of services use would appear in the absence of differences in the distribution of health needs (35). We used an indirect standardization with a probit regression model to calculate the distribution of need-standardized health services utilization, as it was binary (31).

### Second Step: Estimate the Concentration Index and Its Decomposition

We calculated the concentration index to measure socioeconomic inequality in services use (32), ranging from −1 to +1 (36). In the absence of inequalities, the concentration index value should be zero.


(1) $$\begin{array}{r} C=\frac{2}{\mu }cov\left(h,r\right) \end{array}$$

Where *h* is the ranking of health service utilization, *r* is the ranking of the individual by household income, and μ is the mean of health service utilization.

The CI is decomposed into contributions of need factors and non-need factors based on probit regression model (37).


(2) $$\begin{array}{r} y_{i}=\alpha +\underset{j}{\sum }\beta_{j}^{m}x_{ji}+\sum \overset{n}{\underset{k}{\gamma }}z_{ki}+\varepsilon_{i} \end{array}$$

Where *y*_*i*_ is the probability of health services utilization; *x*_*ji*_ are the need factors; *z*_*ki*_ are the non-need factors; $\beta_{j}^{m}$ and $\gamma_{k}^{n}$ are marginal effects of each variable; the α is an intercept; and ε_*i*_ is the error term.

The formula for the decomposition of concentration index:


(3) $$\begin{array}{c} C=\underset{j}{\sum^{\text{​}}}\frac{\beta_{j}^{m}x_{j}}{\mu }C_{j}+\underset{k}{\sum^{\text{​}}}\frac{\gamma_{k}^{m}z_{k}}{\mu }C_{k}+\frac{GC_{\varepsilon }}{\mu } \end{array}$$

Where μ is the mean of *y*; *C*_*j*_ and *C*_*k*_ are the concentration index of *x*_*j*_ and *z*_*k*_; and *GC*_ε_ is the generalized concentration index of the error tem ε (21).

### Third Step: Calculate the HI

We computed the HI by subtracting the contribution of the need factors from the CI. When the HI is positive, individuals with high socioeconomic status are using more services than they need; when it is negative, individuals with low socioeconomic status are using more services than they need (38).


(4) $$\begin{array}{r} HI=CM-CN \end{array}$$

Where CM refers to the CI of actual health service utilization, and CN refers to the CI of the need-expected health service utilization.

All analyses were performed in Stata 16.0.

## Results

### General Data on Health Services Utilization in Heilongjiang Province

The participants were predominantly 45 years of age and above, married, had received primary school or junior middle school education, and employed. Regarding health services utilization, 712 participants had received medical treatment within the previous 2 weeks, while 724 had received inpatient services within the previous year.

The 2-week outpatient rate and annual inpatient rate were 10.74 and 10.93%, respectively. For men and women, both rates increased with age increased (*p* < 0.001). The annual inpatient rate increased at higher economic levels (*p* = 0.002) and was higher among participants who were married (*p* = 0.036). As for education, both rates decreased as educational level increased (*p* < 0.001). As for employment status, the students had the lowest 2-week outpatient rate and annual inpatient rate (*p* < 0.001). Of all participants, 59.12% lived in urban areas. The annual inpatient rate was higher for participants living in rural areas than for those living in urban areas (*p* < 0.001).

Of all participants, 5.79% were not covered by any type of social medical insurance, while 21.49% were covered by Urban Employee Basic Medical Insurance (UEBMI). The remaining 72.72% were covered by urban and rural resident basic medical insurance (URRBMI), Urban Resident Basic Medical Insurance (URBMI), New Rural Cooperative Medical Scheme (NCMS), or other types of social medical insurance schemes. Participants who reported no basic medical insurance showed the lowest two rates; the 2-week outpatient rate and annual inpatient rate were 6.51% (*p* = 0.017) and 3.65% (*p* < 0.001), respectively. Of all participants, 38.15% were covered by critical illness insurance; these participants showed a higher annual inpatient rate when compared to those without critical illness insurance (*p* < 0.001). Meanwhile, 13.02% of all participants were covered by commercial health insurance. There were no statistically significant differences in two rates between participants with and without commercial health insurance (*p* > 0.05). At 96.59%, majority of participants lived within five kilometers to the nearest health facility. The 2-week outpatient rate was higher for participants who lived within five kilometers to the nearest health facility than for those who lived with a distance greater than or equal to this (*p* = 0.025).

The 2-week outpatient rate and the annual inpatient rate also differed based on health status; for the self-assessed health, the 2-week outpatient rate was lowest in the excellent group (5.11%), the annual inpatient rate was lowest in the good group (10.45%). In total, both rates decreased as the self-assessed health status got better statistically significant (*p* < 0.001). Of all participants, 45.60% reported they had chronic disease, while 12.81% reported limitations in their daily activities; in both cases, both rates were, respectively, higher for participants with conditions than for those without (*p* < 0.001) (Table 1).

**Table 1:** Social demographic characteristics, healthcare accessibility, health status, and health services utilization of participants.

**Variables**	**Number**	**(%)**	**Two-week outpatient rate (%)**	***x*** ***^2^/z***	**p**	**Annual inpatient rate (%)**	***x*** ***^2^/z***	**p**
**Gender and age (years)**								
**Men**								
0–14	332	5.01	7.83	−6.12	<0.001	6.33	−10.87	<0.001
15–24	137	2.07	4.38			2.19		
25–34	237	3.58	6.33			2.11		
35–44	511	7.71	6.07			3.52		
45–54	832	12.55	8.41			8.65		
55–64	680	10.26	14.56			15.15		
65–	532	8.03	16.35			24.25		
**Women**								
0–14	345	5.21	6.38	−7.10	<0.001	4.93	−9.12	<0.001
15–24	123	1.86	3.25			6.50		
25–34	263	3.97	9.89			9.51		
35–44	509	7.68	6.09			5.50		
45–54	839	12.66	10.61			7.63		
55–64	733	11.06	13.78			13.37		
65–	554	8.36	18.95			24.01		
**Socioeconomic status**								
**Household income**								
Quintile I	1,329	20.05	11.06	−0.97	0.333	9.18	−3.05	0.002
Quintile II	1,325	19.99	9.89			11.09		
Quintile III	1,375	20.75	10.04			10.11		
Quintile IV	1,273	19.21	10.92			10.84		
Quintile V	1,325	19.99	11.85			13.43		
**Marital status**								
Unmarried and others	1,722	26.74	11.32	0.17	0.679	9.59	4.41	0.036
Married	4,855	73.26	10.65			11.41		
**Education**								
Illiteracy	613	9.25	18.11	5.79	<0.001	17.78	6.30	<0.001
Primary school or junior middle school	4,173	62.97	10.50			11.21		
Senior high school or technical secondary school	1,096	16.54	10.22			7.85		
College, university, or above	745	11.24	6.85			8.19		
**Employment status**								
Employed	3,265	49.27	8.58	82.09	<0.001	6.98	176.32	<0.001
Retired	1,012	15.27	15.22			16.30		
Student	474	7.15	3.59			3.38		
Unemployed and others	1,876	28.31	13.91			16.79		
**Place of residence**								
Urban	3,918	59.12	10.34	1.66	0.198	9.14	31.48	<0.001
Rural	2,709	40.88	11.33			13.51		
**Healthcare accessibility**								
**Type of basic medical insurance scheme**								
UEBMI	1,424	21.49	10.46	8.18	0.017	10.96	22.53	<0.001
URRBMI, URBMI, NCMS, and others	4,819	72.72	11.16			11.50		
None	3,84	5.79	6.51			3.65		
**Critical illness insurance**								
Yes	2,528	38.15	11.23	1.02	0.311	12.70	13.20	<0.001
No	4,099	61.85	10.44			9.83		
**Commercial health insurance**								
Yes	863	13.02	9.85	0.83	0.363	9.62	1.74	0.187
No	5,764	86.98	10.88			11.12		
**Distance to the nearest health facility**								
≤ 5 km	6,401	96.59	10.90	5.05	0.025	10.95	0.13	0.714
>5 km	226	3.41	6.19			10.18		
**Time taken to travel to the nearest health facility**								
<30 min	6,609	99.73	10.76	0.51	0.477	10.94	0.53	0.465
≥30 min	18	0.27	5.56			5.56		
**Health Status**								
**Self-assessed health**								
Very poor	460	6.94	13.70	13.42	<0.001	11.52	13.78	<0.001
Poor	216	3.26	29.17			36.57		
Fair	1,201	18.12	20.73			20.82		
Good	2,067	31.19	9.68			10.45		
Excellent	2,683	40.49	5.11			11.52		
**Chronic disease**								
Yes	3,022	45.60	18.00	305.12	<0.001	18.86	359.59	<0.001
No	3,605	54.40	4.66			4.27		
**limitation of daily activities**								
Yes	849	12.81	26.86	263.57	<0.001	29.33	338.90	<0.001
No	5,778	87.19	8.38			8.22		

*UEBMI, Urban Employee Basic Medical Insurance; URRBMI, Urban and Rural Resident Basic Medical Insurance; URBMI, Urban Resident Basic Medical Insurance; NCMS, The New Rural Cooperative Medical Scheme*.

### Inequality and Inequity in Outpatient and Inpatient Services Utilization

Table 2 presents the results of the CI and HI indices. The CM was positive for both outpatient services utilization (0.0245) and inpatient services utilization (0.0569), which indicated pro-rich inequalities in health services utilization among participants; that is, increased wealth was associated with increased health service utilization. CN was negative for both need-expected outpatient services utilization (−0.0345) and need-expected inpatient services utilization (−0.0701), which indicated pro-poor inequalities in need-expected usage for both outpatient and inpatient services utilization; that is, participants with lower household incomes tended to have greater needs of health services. After taking health services needs into consideration, the HI showed even more pro-rich inequities in both outpatient (HIo = 0.0586) and inpatient services (HIi = 0.1276). In other words, when health services needs were equal, wealthier participants utilized more health services than poorer participants, both in the outpatient and inpatient contexts. Besides, the degree of inequity in inpatient services utilization was 2.18 times that for outpatient services utilization. This indicated that the degree of inequity in inpatient services utilization was higher than that in outpatient services utilization.

**Table 2:** Distribution of actual, need-expected, and need-standardized use of outpatient and inpatient services across household income quintiles.

**Household income**	**Outpatient service use**			**Inpatient service use**		
	**Actual**	**Need-expected**	**Need-standardized**	**Actual**	**Need–expected**	**Need-standardized**
Quintile I (Poorest)	0.1106	0.1191	0.0988	0.0918	0.1324	0.0687
Quintile II	0.0989	0.1116	0.0946	0.1109	0.1182	0.1021
Quintile III	0.1004	0.1037	0.1039	0.1011	0.1060	0.1044
Quintile IV	0.1092	0.1010	0.1155	0.1084	0.0992	0.1186
Quintile V(Richest)	0.1185	0.1009	0.1249	0.1343	0.0939	0.1497
CM/CN/HI	0.0245	−0.0345	0.0586	0.0569	−0.0701	0.1276
SrErr	0.0214	0.0069	0.0201	0.0206	0.0073	0.0189
T	1.14	−4.99	2.92	2.73	−9.57	6.74
*p*	0.253	<0.001	0.004	0.006	<0.001	<0.001

### Distribution of Health Services Utilization Across Household Income Quintiles

Table 2 shows both outpatient services utilization and inpatient services utilization by household income quintiles. There were no statistically significant differences in the actual outpatient services utilization across household income quintiles (*p* = 0.253), while there were statistically significant differences in inpatient services utilization (*p* = 0.006); the actual outpatient and actual inpatient services utilization reported by the richest group were 1.07 times and 1.46 times than those of the poorest, respectively. Quintiles I, II, and III showed underuse in both outpatient services utilization and inpatient services utilization. Quintiles IV and V showed overuse in both outpatient services utilization and inpatient services utilization. Actual outpatient and inpatient services utilization by the poorest group accounted for 92.86 and 69.34% of their need-expected services utilization, respectively. While actual outpatient and inpatient services utilization of the richest group were about 1.17 times and 1.43 times than those of their need-expected health services utilization, respectively. In other words, the poor underused health services, while the rich overused health services.

As reflected by the distribution of need-standardized services utilization, large household income-related inequity was evident in the study population. The gap in health services utilization between the rich and the poor increased after adjusting for health needs; there was a 1.26 times gap (rising from 0.0988 for Quintile I to 0.1249 for Quintile V) for outpatient services use and a 2.18 times gap (rising from 0.0687 for quintile I and 0.1497 for quintile V) for inpatient services use (Table 2). Compared to outpatient services utilization, these results show more obvious inequity in inpatient services utilization.

### Situation That Participants Were Denied Outpatient and Inpatient Services

A small proportion of participants skipped health services they otherwise needed, both in the outpatient and inpatient contexts. Of all participants, 93 refused to use outpatient services over the previous 2 weeks, and 364 refused inpatient services recommended or prescribed by doctors over the past year. Among participants who were denied outpatient services, 50.54% of them were due to financial difficulties. Among participants who were denied inpatient services, 73.63% of them were due to financial difficulties. By the Cochran–Armitage test, we found that a higher socioeconomic status resulted in a lower probability of the participants being denied inpatient services due to financial difficulties (*z* = −2.32, *p* = 0.020) (Table 3).

**Table 3:** Situation that participants were denied outpatient and inpatient services across household income quintiles.

	**I (Poorest)**	**II**	**III**	**IV**	**V (Richest)**	**p**
*N* (%) of denied use of outpatient services (over 2-week)	18 (1.35)	18 (1.36)	17 (1.24)	18 (1.41)	22 (1.66)	0.513
**Reasons for not using outpatient services over 2 weeks (** ***N*** **, %)**						
Financial difficulties	11 (0.83)	9 (0.68)	7 (0.51)	11 (0.86)	9 (0.68)	0.870
Other reasons	7 (0.53)	9 (0.68)	10 (0.73)	7 (0.55)	13 (0.98)	0.275
*N* (%) of refused hospital admission (over past year)	84 (6.32)	74 (5.58)	71 (5.16)	66 (5.18)	69 (5.21)	0.185
**Reasons for not using inpatient services over 1 year (** ***N*** **, %)**						
Financial difficulties	68 (5.12)	54 (4.08)	54 (3.93)	49 (3.85)	43 (3.25)	0.020
Other reasons	16 (1.20)	20 (1.51)	17 (1.24)	17 (1.34)	26 (1.96)	0.193
**Total**	1,329	1,325	1,375	1,273	1,325	

### Decomposition of Inequality of Inpatient Service Utilization

Table 4 shows the results of the decomposition analysis, including each determinant's CI_k_, marginal effect, *p* value of the marginal effect, and contribution to CI. The CI_k_ was employed to describe how each determinant was distributed (ranging from −1 to +1) over the wealth factor. UEBMI coverage (CI_k_ = 0.277) was more concentrated among wealthy participants, while URRBMI, URBMI, NCMS, and other social medical insurance coverage (CI_k_ = −0.078) was more concentrated among poor participants. Critical illness insurance coverage (CI_k_ = −0.040) was also more concentrated among poor participants, while commercial health insurance coverage (CI_k_ = 0.153) was more concentrated among wealthy participants. Finally, poor participants were more likely to live more than five kilometers from the nearest health facility (CI_k_ = −0.077) and needed 30 min or more (CI_k_ = −0.190) to reach them (Table 4).

**Table 4:** Decomposition of household income-related inequalities in outpatient and inpatient services utilization.

	**Outpatient services**				**Inpatient services**			
**Determinants**	**Marginal effects (b_k_)**	**p**		**Contribution to CI (%)**	**Marginal effects (b_k_)**	**p**		**Contribution to CI (%)**
**Gender and age (years)**				**8.16**				**−6.93**
**Men**								
0–14	−0.0547	0.092	0.037	−3.87	−0.0550^*^	0.035	0.036	−1.61
15–24	0.0242	0.606	−0.094	−1.82	−0.0436	0.180	−0.094	1.37
25–34	0.0265	0.569	0.063	2.31	−0.0730^***^	<0.001	0.064	−2.69
35–44	0.0043	0.916	0.118	1.50	−0.0738^***^	<0.001	0.119	−10.94
45–54	0.0019	0.961	0.039	0.36	−0.0645^**^	0.007	0.039	−5.07
55–64	0.0236	0.578	−0.030	−2.75	−0.0571^*^	0.021	−0.030	2.80
65–	0.0141	0.735	−0.129	−5.63	−0.0459	0.084	−0.128	7.65
**Women**								
0–14	−0.0636^*^	0.036	−0.034	4.31	−0.0592^*^	0.021	−0.034	1.69
15–24	Reference							
25–34	0.1144^*^	0.040	0.082	14.32	−0.0286	0.346	0.082	−1.51
35–44	0.0082	0.842	0.097	2.36	−0.0661^**^	0.003	0.097	−7.92
45–54	0.0271	0.520	0.035	4.67	−0.0713^**^	0.002	0.035	−5.11
55–64	0.0138	0.738	−0.062	−3.64	−0.0653^**^	0.005	−0.062	7.19
65–	0.0115	0.780	−0.107	−3.96	−0.0498	0.053	−0.107	7.22
**Socioeconomic status**								
**Household income**				**184.03**				**253.47**
Quintile I	Reference							
Quintile II	−0.0061	0.570	−0.399	18.86	0.0368^**^	0.001	−0.399	−47.53
Quintile III	−0.0013	0.908	0.009	−0.09	0.0437^***^	<0.001	0.009	1.24
Quintile IV	0.0095	0.408	0.408	28.72	0.0566^***^	<0.001	0.408	71.71
Quintile V	0.0221	0.063	0.800	136.54	0.0882^***^	<0.001	0.800	228.05
**Marital status**				**−10.22**				**4.79**
Unmarried and others	Reference							
Married	−0.0139	0.162	0.026	−10.22	0.0155	0.081	0.026	4.79
**Education**				**−26.83**				**11.34**
Illiteracy	Reference							
Primary school or junior middle school	0.0435	0.051	−0.232	−25.05	−0.0245	0.105	−0.232	5.92
Senior high school or technical secondary school	0.0105	0.453	−0.066	−16.91	−0.0248	0.062	−0.066	16.63
College, university, or above	0.0171	0.275	0.139	15.13	−0.0302^*^	0.013	0.139	−11.21
**Employment status**				**32.91**				**−15.74**
Employed	0.0140	0.167	0.037	9.95	−0.0444^***^	<0.001	0.038	−13.34
Retired	0.0197	0.137	0.198	22.93	−0.0042	0.709	0.197	−2.04
Student	0.0022	0.957	0.005	0.03	−0.0616^*^	0.015	0.005	−0.36
Unemployed and others	Reference							
**Place of residence**				**27.21**				**−28.45**
Urban	Reference							
Rural	−0.0159	0.098	−0.108	27.21	0.0398^***^	<0.001	−0.108	−28.45
**Healthcare accessibility**								
**Type of basic medical insurance scheme**				**−17.26**				**26.61**
UEBMI	0.0245	0.243	0.277	56.25	0.0848^**^	0.003	0.276	81.48
URRBMI, URBMI, NCMS, and others	0.0338^*^	0.044	−0.078	−73.51	0.0603^**^	0.001	−0.077	−54.87
None	Reference							
**Critical illness insurance**				**−4.60**				**−0.90**
Yes	0.0079	0.368	−0.040	−4.60	0.0037	0.645	−0.039	−0.90
No	Reference							
**Commercial health insurance**				**20.79**				**7.40**
Yes	0.0270^*^	0.024	0.153	20.79	0.0229^*^	0.042	0.154	7.40
No	Reference							
**Distance to the nearest health facility**				**4.14**				**−0.68**
≤ 5 km	Reference							
>5 km	−0.0409^*^	0.020	−0.077	4.14	0.0159	0.428	−0.078	−0.68
**Time taken to travel to the nearest health facility**				**0.35**				**0.16**
<30 min	Reference							
≥30 min	−0.0176	0.808	−0.190	0.35	−0.0189	0.744	−0.190	0.16
**Health status**								
**Self-assessed health**				**−53.78**				**−22.18**
Very poor	0.0920^**^	0.006	0.008	1.90	0.1001^**^	0.002	0.008	0.84
Poor	0.0990^***^	<0.001	−0.084	−10.48	0.1385^***^	<0.001	−0.084	−6.11
Fair	0.0709^***^	<0.001	−0.086	−42.81	0.0618^***^	<0.001	−0.087	−15.69
Good	0.0258^**^	0.008	−0.008	−2.39	0.0299^**^	0.001	−0.008	−1.22
Excellent	Reference							
**Chronic disease**				**−44.67**				**−16.16**
Yes	0.1126^***^	<0.001	−0.023	−44.67	0.0970^***^	<0.001	−0.023	−16.16
No	Reference							
**limitation of daily activities**				**−39.35**				**−11.61**
Yes	0.0607^***^	<0.001	−0.124	−39.35	0.0428^***^	<0.001	−0.124	−11.61
No	Reference							

* Indicates that the difference was statistically significant ^*^p < 0.05,

** *p < 0.01,*

*** *p < 0.001. Bold values indicate the total contribution to CI of each determinant*.

The marginal effect denotes the association between determinants and health services utilization. A positive marginal effect indicates that the given factor promotes utilization, and vice versa. In addition, the larger the absolute value of *b*_*k*_, the more substantial the association. First, we looked at outpatient services utilization. The *b*_*k*_ showed that participants who were women aged 25–34 years, who were covered by URRBMI, URBMI, NCMS, and other types of basic medical insurance, who had commercial health insurance, who had chronic disease, who reported activity limitation, or who had poorer health status, tended to have increased outpatient services utilization (*p* < 0.05). By contrast, those who were females aged 0–14 years or who lived more than five kilometers from the nearest health facility tended to have decreased outpatient services utilization (*p* < 0.05). We then looked at inpatient services utilization. The *b*_*k*_ showed that participants who had better household incomes, who lived in rural areas, who were covered by basic medical insurance, who had commercial health insurance, who had poorer self-assessed health status, who had chronic disease, or who reported activity limitation, tended to have increased inpatient services utilization (*p* < 0.05). By contrast, participants who were men aged 0–14, 25–34, 35–44, 45–54, and 55–64, who were women aged 0–14, 35–44, 45–54, and 55–64, who had received college, university, or higher education, who were employed, or who were students tended to have decreased inpatient services utilization (*p* < 0.05) (Table 4).

Contributions to CI refer to the relative contributions of each determinant, representing positive or negative contributions to health services utilization inequality. A positive contribution indicates that the corresponding variables aggravate inequality, and vice versa. Household income showed the greatest contribution to inequality in both outpatient and inpatient services utilization, at 184.03 and 253.47%, respectively. For outpatient services, the contributions of self-assessed health, chronic disease, and limitations in daily activities were −53.78, −44.67, and −39.35%, respectively. The contributions of employment status, place of residence, education, and commercial health insurance were 32.91, 27.21, −26.83, and 20.79%, respectively. For inpatient services, the contributions of place of residence, type of basic medical insurance scheme, and self-assessed health were −28.45, 26.61, and −22.18%, respectively. The absolute values of the contributions of the other factors were <20%.

UEBMI made a pro-rich contribution to both outpatient services (56.25%) and inpatient services (81.48%). URRBMI, URBMI, NCMS, and other basic medical insurance made a pro-poor contribution to both outpatient services (−73.51%) and inpatient services (−54.87%), thus indicating a positive role in reducing inequality by enhancing health services utilization among the poor population. Commercial health insurance made a pro-rich contribution to outpatient services (20.79%) and inpatient services (7.40%). Critical illness insurance made a slightly pro-poor contribution to both outpatient services (−4.60%) and inpatient services (−0.90%).

## Discussion

This study analyzed inequities in health services utilization based on data from economically underdeveloped regions in northeast China. We found significant horizontal inequity in which wealthier populations were more likely to access outpatient and inpatient services compared to poor populations.

### The Health System Favors Affluent Populations in Heilongjiang Province

First, the “equal treatment in equal need” principle was not met in the context of health services utilization in Heilongjiang Province. There were pro-rich inequalities in both outpatient and inpatient services when looking at actual use of services (CMo = 0.0245 and CMi = 0.0569). In this regard, poor populations tended to underuse health services, while rich populations tended to overuse them. Among different household income quintiles, the poorest group's actual outpatient and inpatient services utilization accounted for 92.86 and 69.34% of need-expected services utilization, respectively; by contrast, the wealthiest group, respectively, used approximately 1.17 times and 1.43 times that of their need-expected health services utilization. Second, inequity significantly favored the rich at a much higher degree for inpatient services (HIi = 0.1276) than for outpatient services (HIo = 0.0586). These findings clearly show that the health system is more favorable to affluent populations in Heilongjiang province, which is consistent with previous studies from economically underdeveloped regions of China, including those on the middle-aged and elderly in Gansu Province in 2008 (HIo = 0.04626, HIi = 0.03911) (39) and women in Shanxi Province in 2013 (HIo = 0.0493, HIi = 0.0869) (9).The horizontal inequity in this study is also higher than that reported in Kenya (HIo = 0.0096, HIi = 0.0048) (14).

### The Role of Basic Medical Insurance Must Be Strengthened to Reduce Inequality

The UEBMI, NCMS, and URBMI were established in 1998, 2003, and 2007, respectively. These three schemes are separately administered and locally operated to implement different eligibility requirements (employment status, rural household registration, and urban household registration) (40). UEBMI covers urban workers in both public and private sectors; previous studies have reported that UEBMI provides the best health insurance security of the above three types (21, 41). For example, the per capita UEBMI fund was reported at 2573.19 CNY, while those for NCMS and URBMI were reported at 370.59 and 400.48 CNY, respectively (41). Previous studies have shown that the type of medical insurance scheme influences inequality in health services utilization (8, 10). To alleviate inequality between different schemes, China established urban and rural resident basic medical insurance (URRBMI) nationwide in 2016 (42). Against this backdrop, URRBMI was implemented in Heilongjiang Province from December, 2016 (43). As such, this study reported that most participants without UEBMI were covered by URRBMI, with a few participants reporting that they were covered by URBMI, NCMS, or other types of basic medical insurance.

In 2013, 95% of Chinese urban and rural residents were covered by health insurance nationally (44). By 2020, China's health insurance coverage rate had achieved stability at 95% (45). In this study, 5.79% of participants were not covered by any type of social basic medical insurance; these participants also showed a much lower 2-week outpatient rate and annual inpatient rate than others. The average ages for each coverage category were as follows: 52.70 years for UEBMI, 45.79 years for URRBMI, URBMI, NCMS, and other types of basic medical insurance, and 37.62 years for no coverage. We found some evidence of adverse selection for those who were not covered by basic medical insurance. Of participants covered by UEBMI, 22.26% were from the poorest and second-poorest groups, with an average EQ-VAS score of 78.72. For those not covered by basic medical insurance, 45.31% were from the poorest and second-poorest groups, with an average EQ-VAS score of 70.76. Compared to participants who were covered by UEBMI, those without any coverage were younger, poorer, and less healthy. In other words, there was adverse selection, which highlights the need to expand universal health insurance coverage in Heilongjiang Province.

While basic medical insurance had a limited effect in alleviating health services inequalities (−17.26% for outpatient services and 26.61% for inpatient services), this did not counteract the role played by household income in increasing inequalities (184.03% for outpatient services and 253.47% for inpatient services). UEBMI contributed to the wealthy in both outpatient (56.25%) and inpatient (81.48%) services. URRBMI, URBMI, NCMS, and other basic medical insurance made contributions in favor of the poor in both outpatient services (−73.51%) and inpatient services (−54.87%). A previous study similarly reported that UEBMI contributed to the wealthy in both outpatient and inpatient services (10), meanwhile several studies have shown that it works in favor of the wealthy specifically in inpatient services (3, 21, 46). There are two explanations for these findings. First, participants with UEBMI were wealthier than those with URRBMI, URBMI, NCMS, and other basic medical insurance and they could receive high reimbursements that provided by UEBMI. According to Zhao et al. (21), the average actual reimbursement rates of UEBMI and URRMBI were 66.8 and 49.8%, respectively, in 2013. Second, URRBMI, URBMI, NCMS, and other basic medical insurance provide insufficient financial protection. Although the URRBMI, URBMI, and NCMS played positive roles in reducing inequality; this effect was limited. Especially for inpatient services, the positive effect of URRBMI, URBMI, NCMS, and other basic medical insurance (−54.87%) did not counteract the effect of UEBMI (81.48%). Under these conditions, the contribution percentage of basic medical insurance continues to increase inequality in inpatient services. To promote equity in health services utilization, increased efforts should be made to improve the design of URRBMI, such as keeping appropriate premiums, expanding the pooling funds, offering better benefits packages, and ensuring effective reimbursement arrangements. In future, UEBMI and URRBMI can be merged into one new scheme with identical reimbursement policies to ensure equity in health services utilization.

### Critical Illness Insurance Did Slightly Reduce Inequality and Commercial Health Insurance Did Increase Inequality

Critical illness insurance is primarily designed to relieve the economic burden of disease at the individual level, while also reducing inequity and inequality. Previous studies have shown that it reduces financial burden for patients with high medical costs (47, 48), but there is a major lack of evidence on whether it reduces inequality in health services. Therefore, this study adds to the literature by showing that critical illness insurance plays a slightly positive role in reducing inequalities in health services utilization. In this regard, it contributed to the poor in both outpatient (−4.60%) and inpatient (−0.90%) services. In Heilongjiang Province, critical illness insurance is separately administered by local governments, which set their own deductibles therein. For example, in Daqing City (one of the county/city-level units examined in Heilongjiang province), the threshold and top for compensation were 18,000 and 200,000 CNY, respectively, in 2015 (49). Previous studies have also shown that critical health insurance has a limited effect on financial protection in cases of critical illness (47). Based on these findings, the Chinese government must urgently expand the fund investment channel of critical illness insurance, increase critical illness insurance funds, and enhance reimbursement rates to relieve the economic burden of patients and reduce inequity.

In recent decades, the Chinese government has promoted commercial health insurance to handle a variety of difficulties, including an increasing health burden, inequality, and inequity in health services (50). However, very few studies have explored whether commercial health insurance influences inequality in health services. In this study, commercial health insurance had greater effects in promoting outpatient (*b*_*k*_= 0.0270, *p* = 0.024) and inpatient (*b*_*k*_= 0.0229, *p* = 0.042) utilization while contributing to the wealthy in outpatient (20.79%) and inpatient (7.40%) services. There are two explanations for these results. First, participants with commercial health insurance (CI_k_ = 0.153) were more likely to be wealthy, so they had better access to health services. Second, the financial security provided by commercial health insurance promoted health services utilization among the covered participants.

### Populations With Poor Health Status Accessed More Health Services

Chronic diseases, limited daily activities, and poor self-rated health significantly increased the utilization of both outpatient and inpatient services. These factors also played positive roles in reducing inequalities in both outpatient and inpatient services. For example, the concentration percentages for suffering from chronic disease for outpatient and inpatient services were −44.67 and −16.16%, respectively, which were better than those reported in a study that analyzed data from the fourth NHSS, with rates of 73.1 and 9.6%, respectively (3). The rates were also better than those from a previous study that used data from the fifth NHSS to assess women in Shaanxi Province, in which the rates were −12.30 and −2.85%, respectively (9). To some extent, the current results show that health reforms and basic medical insurance reforms implemented by the Chinese government is useful to help protect populations with high health service needs.

### Disparities in Health Resources Hinder Equity

Although China has made great strides in reforming its health system, there are still considerable gaps in healthcare resources and availability between urban and rural areas (51). In fact, this disparities reflect substantial differences in individual socioeconomic conditions (6). In this study, participants in rural areas were poorer than their urban counterparts. Looking specifically at the factor of rural residence, there was an increased inpatient utilization (*b*_*k*_ = 0.0398, *p* < 0.001), decreased inequality in inpatient utilization (−28.45%), and suppressed outpatient utilization, although non-significant (*b*_*k*_ = −0.0159, *p* = 0.098). This may be induced by the fact that compared to urban residents, rural residents were more difficult to access outpatient services; in fact, there is a series of problems, including insufficiency or undersupply of drugs and outpatient services in rural areas. While universal health coverage and universal health insurance coverage have made it easier for rural residents to access inpatient services due to the reimbursement of basic medical insurance. There is evidence that wealthier populations tend to use well-resourced hospitals for outpatient services, while poor populations tend to use poorly resourced primary care institutions for inpatient care (52).

### Limitations to the Study

We acknowledge that the current study has a few limitations. First, the data originated only from the Heilongjiang province and the results of the paper may not be fully applicable to other areas in economically underdeveloped regions of China. Second, the data on household consumption expenditure and health services utilization were all self-reported, which may be prone to potential reporting biases. Nonetheless, self-reported household consumption expenditure and health services utilization have been widely adopted in previous studies in inequity (3, 9, 10). Finally, due to the availability of data, this study is based on cross-sectional data. Analyzing the change of inequity in the health services using longitudinal data is worthy of further studies.

## Conclusions

The “equal treatment in equal need” principle was not met in health services utilization in Heilongjiang province. There were pro-rich inequities in the utilization of both outpatient and inpatient services. Critical illness insurance played a positive role in reducing inequality, while the effect needs to be strengthened. Government should protect the low-income populations from the underutilization of health services and improve the basic medical insurance, critical illness insurance, and social security systems.

## Data Availability Statement

The original contributions presented in the study are included in the article/supplementary materials, further inquiries can be directed to the corresponding authors.

## Author Contributions

XZ, NN, MJ, LG, and QW contributed to conception and design of the study. HZ, BL, LL, HL, KW, YL, and ZK organized the database. XZ, HZ, LS, and YH performed the statistical analysis. XZ wrote the first draft of the manuscript. NN, AR, and HZ wrote sections of the manuscript. All authors contributed to manuscript revision, read, and approved the submitted version.

## Funding

This study was funded by the National Key Social Science Fund of China (Grant No. 19AZD013).

## Conflict of Interest

The authors declare that the research was conducted in the absence of any commercial or financial relationships that could be construed as a potential conflict of interest.

## Publisher's Note

All claims expressed in this article are solely those of the authors and do not necessarily represent those of their affiliated organizations, or those of the publisher, the editors and the reviewers. Any product that may be evaluated in this article, or claim that may be made by its manufacturer, is not guaranteed or endorsed by the publisher.

## Abbreviations

CI: Concentration index
HI: Horizontal inequity index
UEBMI: Urban Employee Basic Medical Insurance
URRBMI: Urban and Rural Resident Basic Medical Insurance
URBMI: Urban Resident Basic Medical Insurance
NCMS: New Rural Cooperative Medical Scheme.
